# Effects of essential mineral elements deficiency and supplementation on serum mineral elements concentration and biochemical parameters in grazing Mongolian sheep

**DOI:** 10.3389/fvets.2023.1214346

**Published:** 2023-07-25

**Authors:** Xiwei Jin, Lingbo Meng, Rui Zhang, Mengjie Tong, Zhi Qi, Lan Mi

**Affiliations:** State Key Laboratory of Reproductive Regulation and Breeding of Grassland Livestock, School of Life Sciences, Inner Mongolia University, Hohhot, China

**Keywords:** biochemical parameter, deficiency, mineral element, serum, sheep, supplementation

## Abstract

Traditional sheep grazing is the pillar industry and the main source of income for local herders in the Inner Mongolia Autonomous Region of China. However, grazing sheep often suffer from mineral deficiency. In the present study, the feeding experiment was performed on 84 grazing Wu Ranke sheep. After being divided into calcium (Ca), zinc (Zn), copper (Cu), cobalt (Co), manganese (Mn), and selenium (Se) treatment groups, they were fed with a mineral deficient diet for 60 days and then a mineral supplement diet for 41 days. Serum samples were collected three times, 10 concentrations of essential mineral elements and 15 concentrations/activity of biochemical parameters were measured to assess the effects of mineral deficiency and supplementation on the physical health of sheep. The results revealed that the sheep showed mineral Ca, Cu, Co, Mn, and Se deficiencies after feeding their respective mineral deficient diet. Deficiency in dietary Ca, Zn, Cu, Co, Mn, and Se may adversely affect the liver, myocardium and pancreas of sheep. The prompt supplementation of dietary Zn, Cu, Co, Mn, and Se may alleviate the damage caused to the liver, myocardium and pancreas, while that of dietary Ca improved energy generation. In conclusion, the adequate supplementation of dietary Ca, Zn, Cu, Co, Mn, and Se is essential for avoiding the impairment caused to the liver, myocardium and pancreas function of sheep by the deficiency in essential dietary minerals.

## 1. Introduction

Sheep and goats are one of the most important livestock providing meat, milk, fur and essential nutrients for humans, including protein, vitamins, fatty acids and minerals (1, 2). Located along the northern border of China, the Inner Mongolia Autonomous Region is the most significant origin of mutton for the country. With more than three-quarters of this region covered by flat natural grassland, traditional ruminant grazing husbandry are the pillar industry and the major source of income for local herdsmen. Thus, the intake of various nutrients by livestock is closely related to the types and amounts of nutrients provided by the grassland in the pasture. However, some research has revealed that pasture is incapable to provide all the nutrients needed for the normal growth and metabolic processes of livestock, especially minerals (3, 4). Various mineral elements are required for the metabolism of livestock through various physiological and biochemical processes. Thus, mineral deficiency often occurs to the livestock if no sufficient mineral elements are provided (5, 6).

In general, the essential mineral nutrients needed by livestock are categorized into macro-elements and micro-elements, with macro-elements including calcium (Ca), phosphorus (P), sodium (Na), sulfur (S), potassium (K), magnesium (Mg), and micro-elements including cobalt (Co), copper (Cu), iodine (I), iron (Fe), zinc (Zn), manganese (Mn), and selenium (Se) (7). The appropriate amounts of these essential mineral elements play a key role in maintaining various biochemical processes for livestock (8). If the sufficient provision of essential mineral nutrients cannot be ensured, the livestock will suffer mineral deficiency (5, 6). Ca is one of the most abundant mineral elements found in livestock, the deficiency of which during the critical growth period often reduces growth rate and hinders skeletal development (9). Sheep and goats often suffer from osteoporosis when Ca is deficient in diets (10). Zn, as the critical component of more than 300 types of metalloenzymes, participates in cellular communication, cellular proliferation and cellular differentiation, playing an important role in the antioxidant system (11). The deficiency of dietary Zn often results in the loss of appetite, metabolic disorders, and the reduction of hemoglobin concentrations (12). Cu is widely found in various enzymes and cofactors, especially in the liver (13, 14). In some research, it is revealed that copper deficiency causes reproductive disorders and alters the immune response of ruminants (15). A persistently low concentration of Cu in the serum of sheep can lead to an increase in the risk of mortality (16). The Co deficiency in ruminants suppresses the synthesis of vitamin B_12_ in the rumen, thus reducing the diversity of rumen microbes (17). Severe Co deficiency leads to liver fat metabolism disorders and muscle lesions in sheep and goats (18, 19). Mn plays a vital role in antioxidants, immunity, and the growth and reproduction of animals (20). The deficiency of Mn in animals causes disruption to bone-regulating hormones and reduces bone metabolic indexes in serum (21). Early studies have shown that feeding Mn deficiency diets to ruminants reduced conception rates (22). The adequate provision of Se, which is an important component of selenoprotein, is essential for bone metabolism, immunity and endocrine (5). The ruminants with Se deficiency are prone to white muscle disease and immunosuppression, especially for junior ruminants (23).

There are many studies revealing that the grazing sheep farmed on the grassland in many parts of China and other countries around the world are often deficient in various mineral elements including Ca, Zn, Cu, Co, Mn, Se (24–26). Similarly, some studies have shown that soils, pastures and grazing sheep in the Inner Mongolia Autonomous Region are also deficient in Ca, Zn, Cu, Co, Mn, and Se (27–30). Up to now, it remains unclear how the lack of dietary minerals is reflected in the serum and affects the health of grazing Mongolian sheep. Total-reflection X-Ray Fluorescence (TXRF) is an analytical technique that is applicable to detect various elements simultaneously at trace level (31). Currently, TXRF technique has been widely used in bioscience due to its fastness, precision and accuracy. Mineral nutrient deficiency can be accurately diagnosed through serum tests, while biochemical parameters in serum can effectively assess the tissue damage, which reflects the health status of animals (32). Ca, Zn, Cu, Co, Mn, and Se are susceptible to deficiency in grazing sheep, due to insufficient mineral nutrients from pasture under the natural grassland grazing ecosystem. Therefore, an approval was granted for the feeding experiments on a total of 84 grazing Wu Ranke sheep divided into 6 treatment groups fed with Ca, Zn, Cu, Co, Mn, and Se deficient multi-nutrient salt diet, respectively, for 60 consecutive days and then supplemented multi-nutrient salt diet, respectively, for 41 consecutive days. The essential mineral elements and biochemical parameters in the serum of 84 grazing Mongolian sheep were detected to explore the impact of respective diets on animal health. Aiming to provide guidance for the rational mineral supplementation of grazing sheep.

## 2. Materials and methods

### 2.1. Experimental design

All the procedures of animal experiments were approved and conducted in strict accordance with the requirements set out by the Inner Mongolia University Animal Care and Use Committee (IMU-2020-sheep-040). A total of 84 4-month-old female grazing Wu Ranke sheep were purchased from Abaga Banner, Xilin Gol League, Inner Mongolia Autonomous Region, China. All of the sheep were housed and fed individually with native grasses as roughage, crushed oats as concentrate, and multi-nutrient salts as recommended by the National Research Council (NRC) (33).

**Figure 1:**
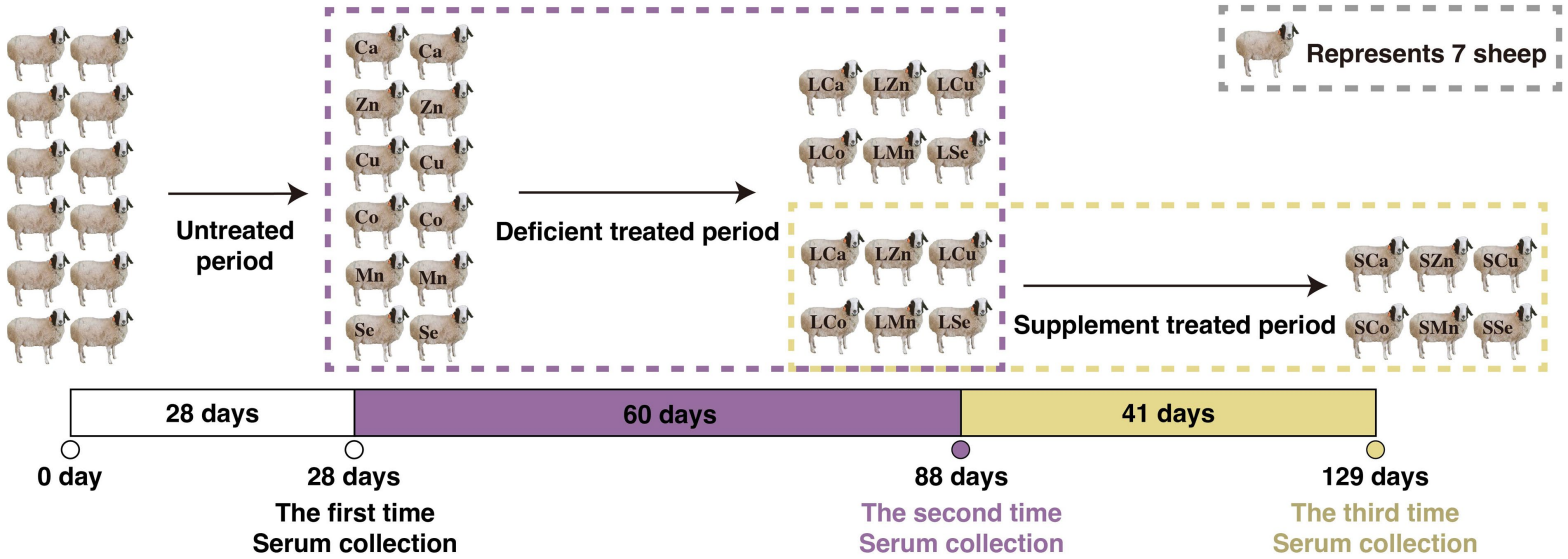
Feeding experimental design of the respective calcium (Ca), zinc (Zn), copper (Cu), cobalt (Co), manganese (Mn), selenium (Se) deficiency and supplement treatments in the 84 Mongolian Wu Ranke sheep.

During the 28-day pre-feeding period, all the Mongolian Wu Ranke sheep were fed with crushed oats and native grasses. Afterward, these 84 Mongolian Wu Ranke sheep were equally divided into Ca (LCa), Zn (LZn), Cu (LCu), Co (LCo), Mn (LMn), and Se (LSe) deficient groups on a random basis and fed with Ca, Zn, Cu, Co, Mn, and Se deficient multi-nutrient salt diet, respectively, for 60 consecutive days. Then, 7 sheep were randomly selected out of each group to continue feeding on Ca, Zn, Cu, Co, Mn, and Se supplemented multi-nutrient salt diet, respectively, for 41 consecutive days, with them denoted as Ca (SCa), Zn (SZn), Cu (SCu), Co (SCo), Mn (SMn), and Se (SSe) supplemented group (Figure 1). The formulation of the multi-nutrient salts for each group is detailed in Tables 1, 2.

**Table 1:** Formulation of the multi-nutrient salts for grazing Mongolian Wu Ranke sheep (Ca, Zn, Cu, Co, Mn, and Se deficient groups).

Items	LCa	LZn	LCu	LCo	LMn	LSe
KCl (g/d)	11.5	11.5	11.5	11.5	11.5	11.5
Na_2_SO_4_ (g/d)	9	9	9	9	9	9
NH_4_H_2_PO_4_ (g/d)	7.4	7.4	7.4	7.4	7.4	7.4
NaCl (g/d)	5	5	5	5	5	5
MgO (g/d)	1.7	1.7	1.7	1.7	1.7	1.7
FeSO_4_ (mg/d)	200	200	200	200	200	200
Ca (IO_3_)_2_ (mg/d)	1.4	1.4	1.4	1.4	1.4	1.4
CaCO_3_ (g/d)	0	10	10	10	10	10
ZnSO_4_ (mg/d)	132	0	132	132	132	132
MnSO_4_ (mg/d)	92	92	92	92	0	92
CuSO_4_ (mg/d)	36	36	0	36	36	36
CoSO_4_ (mg/d)	1.2	1.2	1.2	0	1.2	1.2
Na_2_SeO_3_ (mg/d)	1	1	1	1	1	0

**Table 2:** Formulation of the multi-nutrient salts for grazing Mongolian Wu Ranke sheep (Ca, Zn, Cu, Co, Mn, and Se supplement groups).

Items	SCa	SZn	SCu	SCo	SMn	SSe
KCl (g/d)	11.5	11.5	11.5	11.5	11.5	11.5
Na_2_SO_4_ (g/d)	9	9	9	9	9	9
NH_4_H_2_PO_4_ (g/d)	7.4	7.4	7.4	7.4	7.4	7.4
NaCl (g/d)	5	5	5	5	5	5
MgO (g/d)	1.7	1.7	1.7	1.7	1.7	1.7
FeSO_4_ (mg/d)	200	200	200	200	200	200
Ca (IO_3_)_2_ (mg/d)	1.4	1.4	1.4	1.4	1.4	1.4
CaCO_3_ (g/d)	20	10	10	10	10	10
ZnSO_4_ (mg/d)	132	396	132	132	132	132
MnSO_4_ (mg/d)	92	92	92	92	276	92
CuSO_4_ (mg/d)	36	36	108	36	36	36
CoSO_4_ (mg/d)	1.2	1.2	1.2	3.6	1.2	1.2
Na_2_SeO_3_ (mg/d)	1	1	1	1	1	3

### 2.2. Sample collection

The blood samples of each Mongolian Wu Ranke sheep were collected on day 28 (the end of the pre-feeding period, *n* = 14 for each group), on day 88 (the end of the feeding deficient multi-nutrient salts period, *n* = 14 for each group), and on day 129 (the end of the feeding supplement multi-nutrient salts period, *n* = 7 for each group) of the feeding period, respectively. At 8:00 a.m., Jugular vein blood was collected from the Mongolian Wu Ranke sheep using serum separator vacuum tubes following 12 h fast. Then, the blood was centrifuged at 3,000 rpm for 10 min to obtain serum samples and stored in microtubes at −20°C for later laboratory analysis.

### 2.3. Detection of essential mineral elements in serum

#### 2.3.1. Digestion of serum samples by microwave

The serum samples were digested by a microwave digester (REVO, Labtech, Beijing, China). 300 uL of each serum sample was added to 6 mL 65% HNO_3_, and then digested in strict accordance with procedures shown in Supplementary Table S1. Each of the digested samples was filled to 10 mL by ddH_2_O as the sample to be measured (34).

#### 2.3.2. Measurement of the serum essential mineral elements

Firstly, 200 uL of the digested sample was mixed with 2 uL of gallium (Ga) solution (1,000 mg/L) and 20 uL of polyvinyl alcohol solution (0.3 g/L) to obtain the mixed sample. After thorough homogenization, 10 uL of the mixed sample was transferred to a number of quartz glass sample carriers and dried in the oven at 50°C (35). The concentration of 10 essential mineral elements including P, S, K, Ca, Mn, Fe, Co, Cu, Zn, and Se was measured by the TXRF spectrometer (S4 T-STAR, Bruker Nano GmbH, Berlin, Germany) fitted with a molybdenum (Mo) X-ray tube. A voltage of 50 kV and a current of 1,000 uA were applied to excite the serum sample. The measurement lasted 300 s for each sample (36).

#### 2.3.3. Establishment of the standard curves

The element standard curves were established using the 1,000 mg/L elements mixed standard solution of K, Ca, chromium (Cr), Mn, Fe, Co, nickel (Ni), Cu, Zn, Se, cadmium (Cd), aluminum (Al), boron (B), barium (Ba), zirconium (Zr), lithium (Li), Mg, Na, plumbum (Pb) (BWB2308-2016, Weiye Metrology and Technology Research Group Co., Ltd., Beijing, China), 1,000 mg/L P standard solution (GSB04-1741-2004, Guobiao (Beijing) Testing & Certification Co., Ltd., Beijing, China) and 1,000 mg/L S standard solution (GSB04-1773-2004, Guobiao (Beijing) Testing & Certification Co., Ltd., Beijing, China) were used to establish the element standard curves. The above standard solutions were diluted to 800 mg/L, 500 mg/L, 400 mg/L, 200 mg/L, 160 mg/L, 120 mg/L, 60 mg/L, 30 mg/L, 24 mg/L, 12 mg/L, 6 mg/L, 3 mg/L, 1.2 mg/L, 0.3 mg/L, 0.03 mg/L by appropriate dissolving medium in steps, respectively. The standard solutions of those 16 different concentrations were digested by the microwave digester strictly according to the same procedure as serum samples.

Each concentration of the digested standard solution was measured by the TXRF spectrometer, with the measurement result as the concentration of the respective element standard solution. Both the actual and measured concentrations of the standard solution were fitted to the standard curve of each element. All the results of serum element measurement were calibrated with the standard curve.

### 2.4. Measurement of serum biochemical parameters

The automatic biochemical analyzer (BS-240VET, Mindray, Beijing, China) was used to measure the 8 serum biochemical parameters concentration of triglycerides (TG), UREA, total bilirubin (TBil), total protein (TP), total cholesterol (TC), glucose (GLU), creatinine (CREA), albumin (ALB) and the 7 serum biochemical parameters activity of alanine aminotransferase (ALT), γ-glutamyl transferase (γ-GT), aspartate transaminase (AST), alkaline phosphatase (ALP), lipase (LIP), α-amylase (α-AMY) and creatine kinase (CK). Before measurement, the instruments were calibrated by standard (37).

### 2.5. Statistical analysis

The normality of data distribution was checked by the Shapiro-Wilk test (38). *T*-test was performed to determine the significant difference between the pre-feeding period, the feeding deficient multi-nutrient salts period, and the feeding supplement multi-nutrient salts period of the measured serum essential mineral elements and biochemical parameters. The Paired sample *t*-test was carried out using GraphPad Prism (v.9.3.1). The test results were expressed as mean ± standard error (SE), and the differences of *p* < 0.05 was considered as significant. The results were visualized by R (v.4.1.2). Vector drawing was performed using Adobe Illustrator 2020.

## 3. Results

### 3.1. Measurement of serum essential mineral elements

#### 3.1.1. Concentration of serum essential mineral elements in Ca treatment group

**Figure 2:**
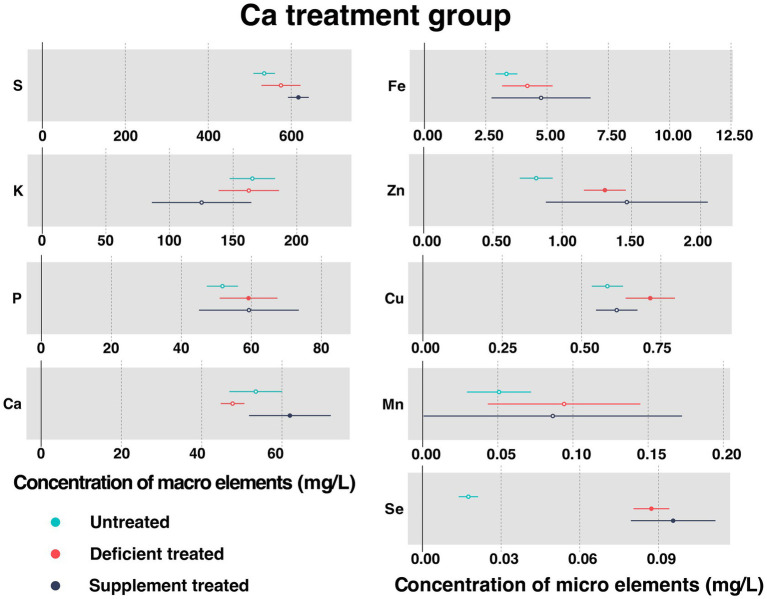
The concentration of measured serum essential mineral elements in Ca treatment group. The paired samples *t*-test was performed to determine the significant difference between untreated (*n* = 14) and deficient treatment (*n* = 14), between deficient treatment (*n* = 7) and supplement treatment (*n* = 7). The differences of *p* < 0.05 were considered as significant. The solid point in the figure represents *p* < 0.05 and the hollow point represents *p* > 0.05. The lines on either side of the point represent the standard error for the data.

Figure 2 shows the concentration of measured serum essential mineral elements in Ca treatment group. Ca deficiency treatment had no effect on the concentration of serum Ca when compared with the untreated. Serum Ca concentration was significantly increased after dietary Ca supplementation compared with Ca deficiency period (61.98 ± 5.20 mg/L vs. 47.71 ± 1.35 mg/L, *p* < 0.05).

Ca deficiency treatment made no difference to the concentration of serum S, K, Fe, and Mn compared with the untreated. In addition, the concentration of S, Fe, and Mn showed an increasing trend while that of K exhibited a decreasing trend. Ca deficiency treatment led to a significant increase in the concentration of P (59.17 ± 4.21 mg/L vs. 51.71 ± 2.27 mg/L, *p* < 0.05), Zn (1.31 ± 0.077 mg/L vs. 0.81 ± 0.060 mg/L, *p* < 0.05), Cu (0.77 ± 0.033 mg/L vs. 0.59 ± 0.037 mg/L, *p* < 0.05), and Se (0.087 ± 0.0035 mg/L vs. 0.018 ± 0.0019 mg/L, *p* < 0.05) when compared with the untreated.

Ca supplement treatment had no impact on the serum concentration of K, P, Fe, Zn, Cu, and Mn in comparison with the Ca deficiency treatment. In addition, the concentration of P, Fe, and Zn exhibited an increasing trend while that of K, Cu, and Mn displayed a decreasing trend. Ca supplement treatment caused a significant increase in the concentration of S (617.27 ± 12.76 mg/L vs. 502.22 ± 15.07 mg/L, *p* < 0.05) and Se (0.096 ± 0.0082 mg/L vs. 0.082 ± 0.0055 mg/L, *p* < 0.05) when compared with Ca deficiency treatment.

#### 3.1.2. Concentration of serum essential mineral elements in Zn treatment group

**Figure 3:**
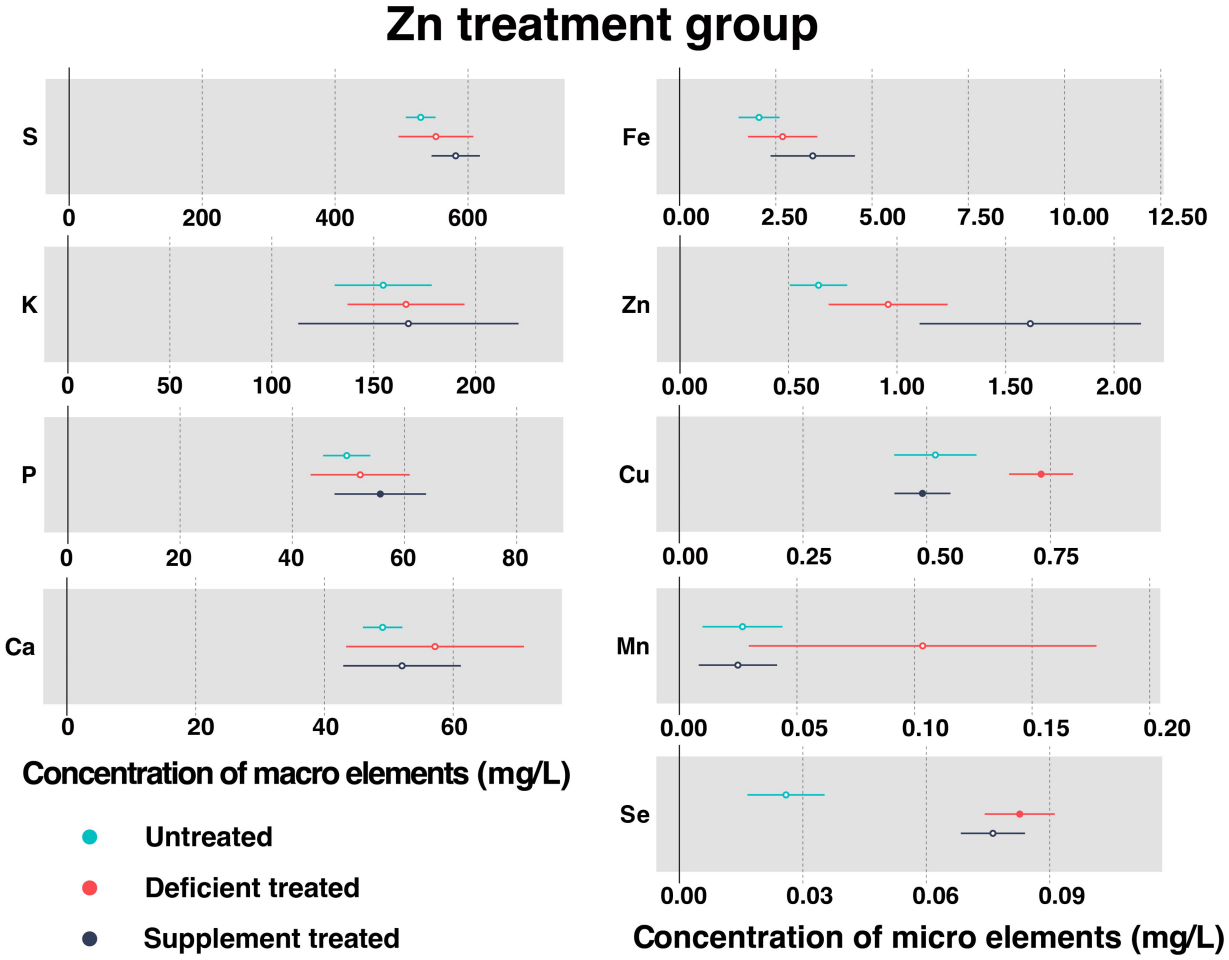
The concentration of measured serum essential mineral elements in Zn treatment group. The paired samples *t*-test was performed to determine the significant difference between untreated (*n* = 14) and deficient treatment (*n* = 14), between deficient treatment (*n* = 7) and supplement treatment (*n* = 7). The differences of *p* < 0.05 were considered as significant. The solid point in the figure represents *p* < 0.05 and the hollow point represents *p* > 0.05. The lines on either side of the point represent the standard error for the data.

Figure 3 shows the concentration of measured serum essential mineral elements in Zn treatment group. Both Zn deficiency treatment and Zn supplement treatment made no difference to the concentration of serum Zn.

After Zn deficiency treatment, the concentration of serum Cu (0.73 ± 0.033 mg/L vs. 0.52 ± 0.042 mg/L, *p* < 0.05) and Se (0.083 ± 0.0043 mg/L vs. 0.026 ± 0.0048 mg/L, *p* < 0.05) was significantly improved when compared with the untreated, while that of serum S, K, P, Ca, Fe, and Mn showed an increasing trend.

In comparison with the Zn deficiency treatment, Zn supplement treatment had no effect on the concentration of serum S, K, Ca, Fe, Mn, and Se. The concentration of serum S, K and Fe showed an increasing trend while that of Ca, Mn and Se exhibited a decreasing trend. Zn supplement treatment significantly increased the concentration of serum P (55.69 ± 4.16 mg/L vs. 38.59 ± 2.88 mg/L, *p* < 0.05) but reduced that of serum Cu (0.49 ± 0.029 mg/L vs. 0.74 ± 0.044 mg/L, *p* < 0.05).

#### 3.1.3. Concentration of serum essential mineral elements in Cu treatment group

**Figure 4:**
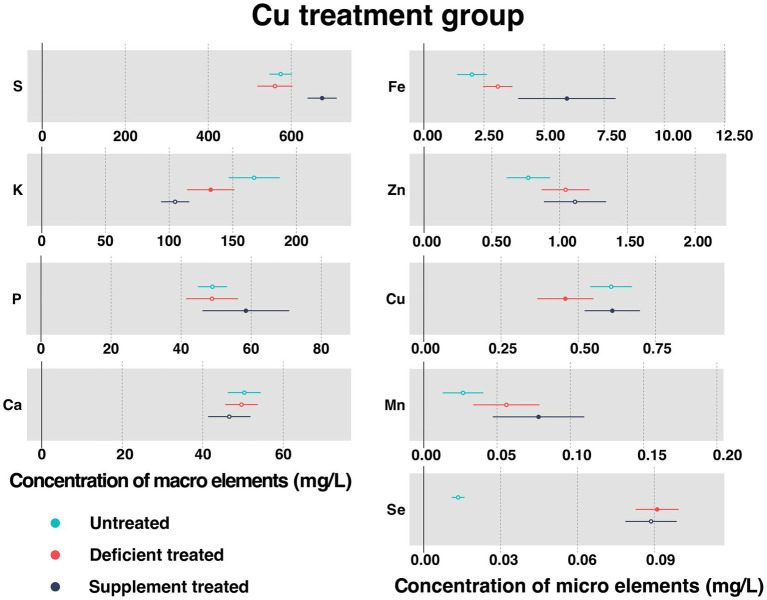
The concentration of measured serum essential mineral elements in Cu treatment group. The paired samples *t*-test was performed to determine the significant difference between untreated (*n* = 14) and deficient treatment (*n* = 14), between deficient treatment (*n* = 7) and supplement treatment (*n* = 7). The differences of *p* < 0.05 were considered as significant. The solid point in the figure represents *p* < 0.05 and the hollow point represents *p* > 0.05. The lines on either side of the point represent the standard error for the data.

Figure 4 shows the concentration of measured serum essential mineral elements in Cu treatment group. Cu deficiency treatment contributed to a sharp decline in serum Cu concentration (0.46 ± 0.046 mg/L vs. 0.61 ± 0.034 mg/L, *p* < 0.05). Followed by the Cu supplementation treatment, serum Cu concentration showed a significant increase in serum (0.61 ± 0.046 mg/L vs. 0.52 ± 0.062 mg/L, *p* < 0.05).

Compared with the untreated, there were no significant differences observed in the concentration of serum S, P, Ca, Fe, Zn, and Mn after the Cu deficiency treatment. Specifically, the concentration of Fe, Zn, and Mn showed an increasing trend but that of the S, P, and Ca showed a decreasing trend. After Cu deficiency treatment, Se concentration (0.091 ± 0.0043 mg/L vs. 0.013 ± 0.0013 mg/L, *p* < 0.05) increased significantly but K concentration (132.65 ± 9.49 mg/L vs. 166.80 ± 10.25 mg/L, *p* < 0.05) was reduced sharply compared to the untreated period.

Following the Cu supplement treatment, the concentration of serum K, Ca, Zn, and Se showed no significant difference. Except for Zn that exhibited an increasing trend, all the other measured elements showed a decreasing trend. Compared with the Cu deficiency treatment, the concentration of S (674.50 ± 17.99 mg/L vs. 513.77 ± 34.059 mg/L, *p* < 0.05), P (58.48 ± 6.32 mg/L vs. 38.05 ± 3.16 mg/L, *p* < 0.05), Fe (5.95 ± 1.034 mg/L vs. 2.16 ± 0.24 mg/L, *p* < 0.05), and Mn (0.078 ± 0.016 mg/L vs. 0.053 ± 0.021 mg/L, *p* < 0.05) increased significantly after dietary Cu supplementation.

#### 3.1.4. Concentration of serum essential mineral elements in Co treatment group

**Figure 5:**
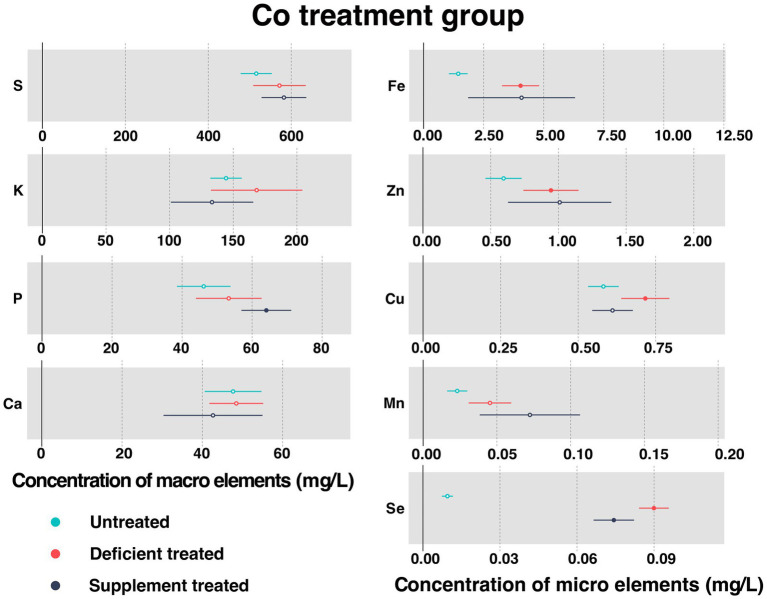
The concentration of measured serum essential mineral elements in Co treatment group. The paired samples *t*-test was performed to determine the significant difference between untreated (*n* = 14) and deficient treatment (*n* = 14), between deficient treatment (*n* = 7) and supplement treatment (*n* = 7). The differences of *p* < 0.05 were considered as significant. The solid point in the figure represents *p* < 0.05 and the hollow point represents *p* > 0.05. The lines on either side of the point represent the standard error for the data.

Figure 5 shows the concentration of measured serum essential mineral elements in Co treatment group. Co was not detected in the Mongolian Wu Ranke sheep studied regardless of the treatment, which may be due to the serum Co concentration falling below the lower limit of detection.

Despite no significant increase in the concentration of serum S, K, P, Ca, and Mn after Co deficiency treatment, they all showed an increasing trend. The concentration of Fe (4.038 ± 0.39 mg/L vs. 1.44 ± 0.20 mg/L, *p* < 0.05), Zn (0.94 ± 0.10 mg/L vs. 0.60 ± 0.068 mg/L, *p* < 0.05), Cu (0.72 ± 0.040 mg/L vs. 0.58 ± 0.025 mg/L, *p* < 0.05), and Se (0.090 ± 0.0029 mg/L vs. 0.0095 ± 0.0011 mg/L, *p* < 0.05) increased significantly after Co deficiency treatment when compared with the untreated.

After Co supplement treatment, there was no effect observed on the concentration of S, K, Ca, Fe, Zn, Cu, and Mn. To be specific, the concentration of S, Fe, Zn, and Mn exhibited an increasing trend but that of K, Ca, and Cu showed a decreasing trend. Following the Co supplement treatment, the concentration of P (64.07 ± 3.63 mg/L vs. 39.04 ± 5.05 mg/L, *p* < 0.05) increased significantly but that of Se (0.074 ± 0.0040 mg/L vs. 0.088 ± 0.0057 mg/L, *p* < 0.05) decreased significantly compared with Co deficiency treatment.

#### 3.1.5. Concentration of serum essential mineral elements in Mn treatment group

**Figure 6:**
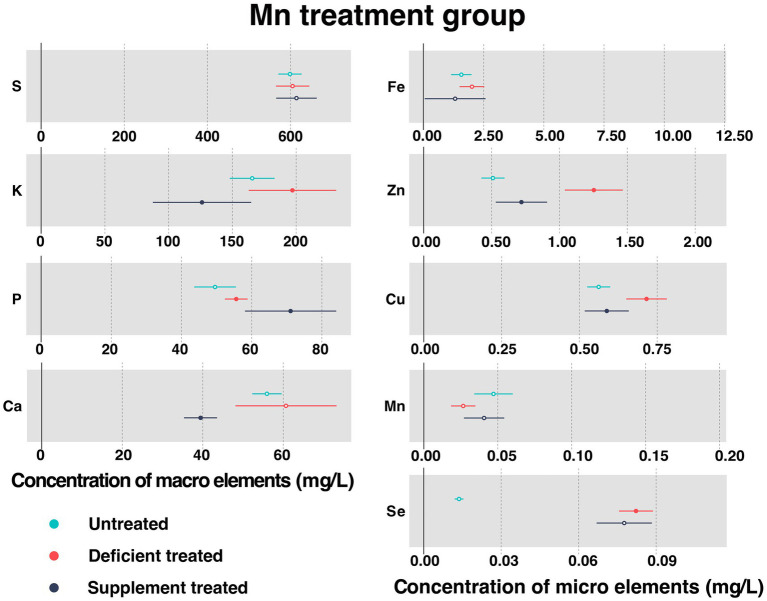
The concentration of measured serum essential mineral elements in Mn treatment group. The paired samples *t*-test was performed to determine the significant difference between untreated (*n* = 14) and deficient treatment (*n* = 14), between deficient treatment (*n* = 7) and supplement treatment (*n* = 7). The differences of *p* < 0.05 were considered as significant. The solid point in the figure represents *p* < 0.05 and the hollow point represents *p* > 0.05. The lines on either side of the point represent the standard error for the data.

Figure 6 shows the concentration of measured serum essential mineral elements in Mn treatment group. Mn deficiency treatment and Mn supplement treatment made no significant difference to the concentration of serum Mn.

Due to Mn deficiency treatment, there was a decreasing trend shown by the concentration of serum Mn. Despite no significant difference caused by the concentration of S, Ca, and Fe, they still showed an increasing trend. The concentration of K (197.08 ± 17.50 mg/L vs. 165.52 ± 8.95 mg/L, *p* < 0.05), P (55.61 ± 1.65 mg/L vs. 49.57 ± 3.03 mg/L, *p* < 0.05), Zn (1.25 ± 0.11 mg/L vs. 0.51 ± 0.043 mg/L, *p* < 0.05), Cu (0.72 ± 0.033 mg/L vs. 0.56 ± 0.019 mg/L, *p* < 0.05) and Se (0.082 ± 0.0034 mg/L vs. 0.014 ± 0.00086 mg/L, *p* < 0.05) increased significantly compared with the untreated.

After the Mn supplement treatment, the concentration of S exhibited an increasing trend but that of Fe and Se showed a decreasing trend compared with the Mn deficiency treatment. After Mn supplement treatment, the concentration of P (71.099 ± 6.63 mg/L vs. 52.28 ± 2.50 mg/L, *p* < 0.05) rose sharply but that of K (126.17 ± 19.66 mg/L vs. 147.096 ± 17.58 mg/L, *p* < 0.05), Ca (39.45 ± 2.09 mg/L vs. 76.58 ± 10.65 mg/L, *p* < 0.05), Zn (0.72 ± 0.096 mg/L vs. 1.47 ± 0.17 mg/L, *p* < 0.05), and Cu (0.59 ± 0.036 mg/L vs. 0.76 ± 0.049 mg/L, *p* < 0.05) plunged when compared with the Mn deficiency treatment.

#### 3.1.6. Concentration of serum essential mineral elements in Se treatment group

**Figure 7:**
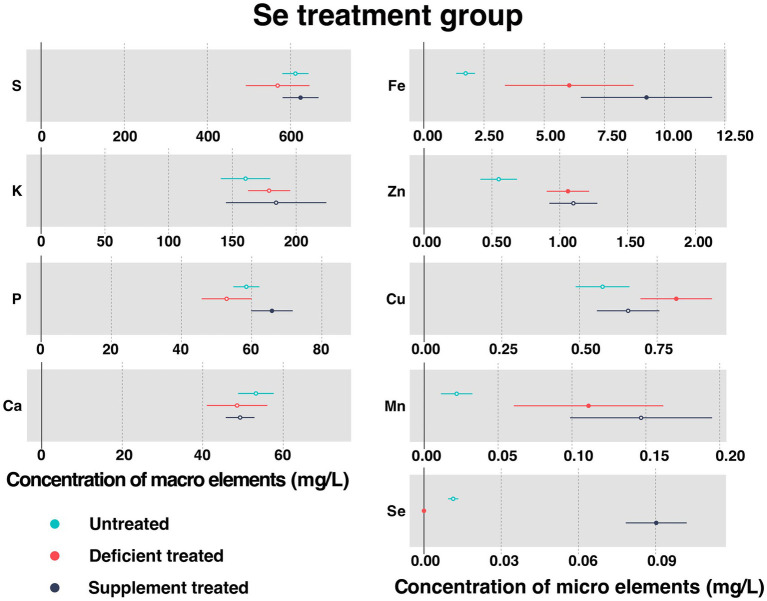
The concentration of measured serum essential mineral elements in Se treatment group. The paired samples *t*-test was performed to determine the significant difference between untreated (*n* = 14) and deficient treatment (*n* = 14), between deficient treatment (*n* = 7) and supplement treatment (*n* = 7). The differences of *p* < 0.05 were considered as significant. The solid point in the figure represents *p* < 0.05 and the hollow point represents *p* > 0.05. The lines on either side of the point represent the standard error for the data.

Figure 7 shows the concentration of measured serum essential mineral elements in Se treatment group. Se deficiency treatment resulted in a significant decrease in the concentration of serum Se (0 ± 0 mg/L vs. 0.11 ± 0.010 mg/L, *p* < 0.05). A sharp rise in the level of serum Se concentration occurred after the Se supplement treatment (0.090 ± 0.0060 mg/L vs. 0 ± 0 mg/L, *p* < 0.05).

Se deficiency treatment had no effect on the concentration of S, K, P, and Ca. In addition, the concentration of K displayed an increasing trend but that of S, P, and Ca exhibited a decreasing trend when compared with the untreated period. Se deficiency treatment caused a significant increase in the concentration of Fe (6.04 ± 1.36 mg/L vs. 1.74 ± 0.20 mg/L, *p* < 0.05), Zn (1.06 ± 0.080 mg/L vs. 0.55 ± 0.069 mg/L, *p* < 0.05), Cu (0.81 ± 0.059 mg/L vs. 0.57 ± 0.044 mg/L, *p* < 0.05), and Mn (0.11 ± 0.026 mg/L vs. 0.022 ± 0.0054 mg/L, *p* < 0.05).

After Se supplement treatment, the concentration of K, Ca, Zn, and Mn showed an increasing trend but that of Cu displayed a decreasing trend. During this period, the concentration of S (624.13 ± 22.10 mg/L vs. 493.02 ± 57.43 mg/L, *p* < 0.05), P (65.79 ± 3.02 mg/L vs. 42.58 ± 3.39 mg/L, *p* < 0.05) and Fe (9.25 ± 1.39 mg/L vs. 3.11 ± 0.41 mg/L, *p* < 0.05) rose sharply compared with the Se deficiency treatment.

### 3.2. Measurement of the serum biochemical parameters

#### 3.2.1. Concentration and activity of serum biochemical parameters in Ca treatment group

**Figure 8:**
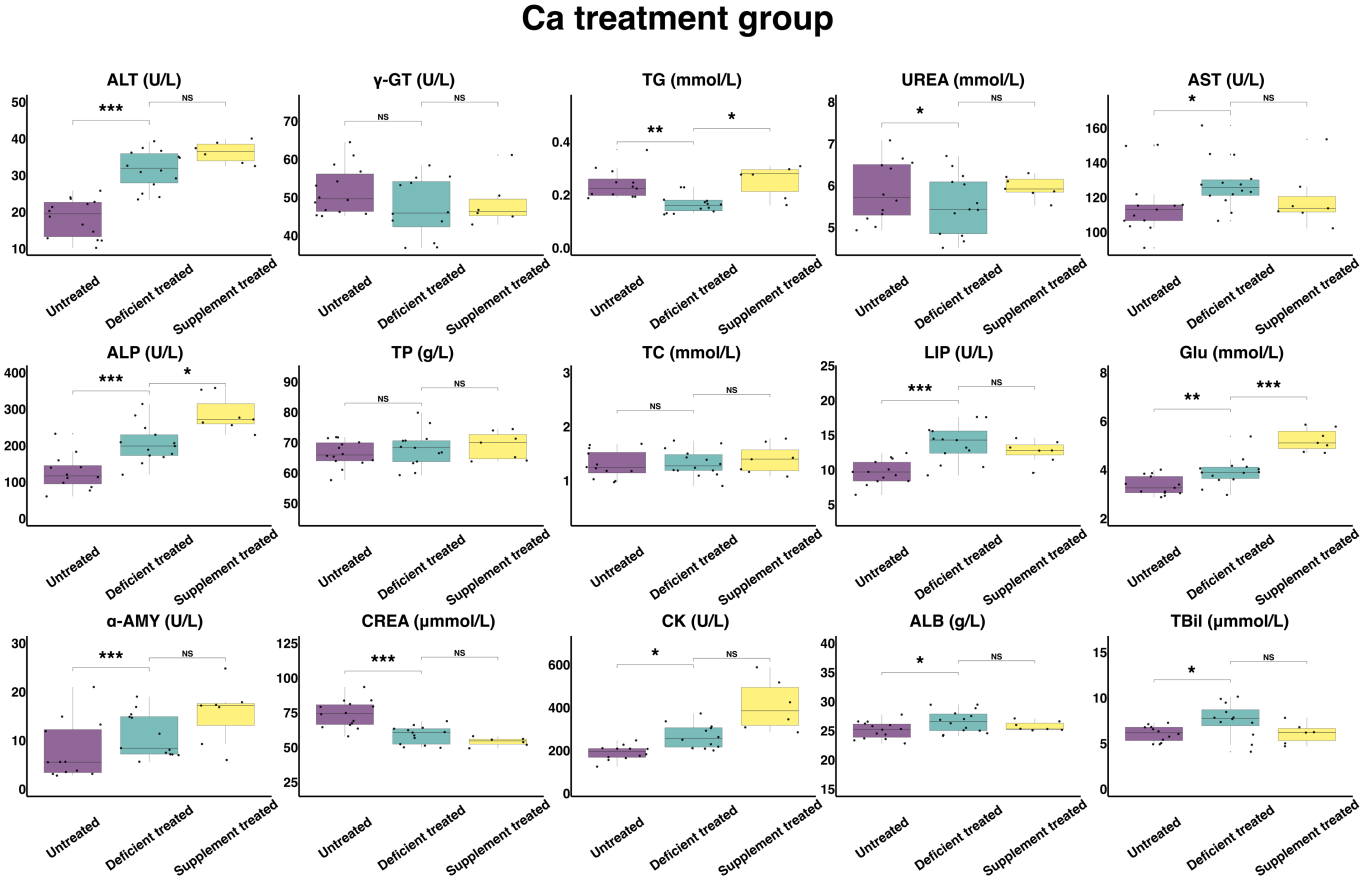
The results of the serum biochemical parameters concentration and the serum biochemical parameters activity in Ca treatment group. ^NS^*p* > 0.05, *p < 0.05, **p < 0.01, ***p < 0.001.

Figure 8 shows the results of serum biochemical parameters concentration and serum biochemical parameters activity in Ca treatment group. After Ca deficiency treatment, there were no significant differences observed in serum TP, TC concentration and γ-GT activity compared with the untreated period. After Ca deficiency treatment, there was a significant increase in the concentration of GLU (3.93 ± 1.05 mmol/L vs. 3.36 ± 0.93 mmol/L, *p* < 0.05), ALB (26.59 ± 7.11 g/L vs. 25.14 ± 6.72 g/L, *p* < 0.05) and TBil (7.58 ± 2.19 umol/L vs. 6.08 ± 1.76 umol/L, *p* < 0.05) and the activity of ALT (31.69 ± 8.47 U/L vs. 18.39 ± 4.92 U/L, *p* < 0.05), AST (127.94 ± 34.19 U/L vs. 115.45 ± 32.02 U/L), ALP (205.18 ± 56.91 U/L vs. 126.81 ± 36.61 U/L, *p* < 0.05), LIP (13.72 ± 3.80 U/L vs. 9.65 ± 2.68 U/L, *p* < 0.05), α-AMY (11.02 ± 3.06 U/L vs. 7.84 ± 2.26 U/L, *p* < 0.05) and CK (266.38 ± 76.90 U/L vs. 190.11 ± 54.88 U/L, *p* < 0.05). Ca deficiency treatment significantly decreased the concentration of the TG (0.17 ± 0.046 mmol/L vs. 0.24 ± 0.070 mmol/L, *p* < 0.05), UREA (5.55 ± 1.54 mmol/L vs. 5.87 ± 1.70 mmol/L, *p* < 0.05) and CREA (58.92 ± 16.34 umol/L vs. 74.18 ± 19.83 umol/L, *p* < 0.05).

There were no significant differences found after Ca supplement treatment in the concentration of serum ALB, TBil, UREA, TP, TC and CREA and the activity of ALT, AST, LIP, α-AMY, CK, and γ-GT when compared with the Ca deficiency treatment. The concentration of TG (0.25 ± 0.10 mmol/L vs. 0.18 ± 0.066 mmol/L, *p* < 0.05) and GLU (5.23 ± 1.98 mmol/L vs. 3.62 ± 1.37 mmol/L, *p* < 0.05) and the activity of ALP (286.84 ± 108.42 U/L vs. 241.29 ± 91.20 U/L, *p* < 0.05) were significantly higher in comparison with the Ca deficiency treatment.

#### 3.2.2. Concentration and activity of serum biochemical parameters in Zn treatment group

**Figure 9:**
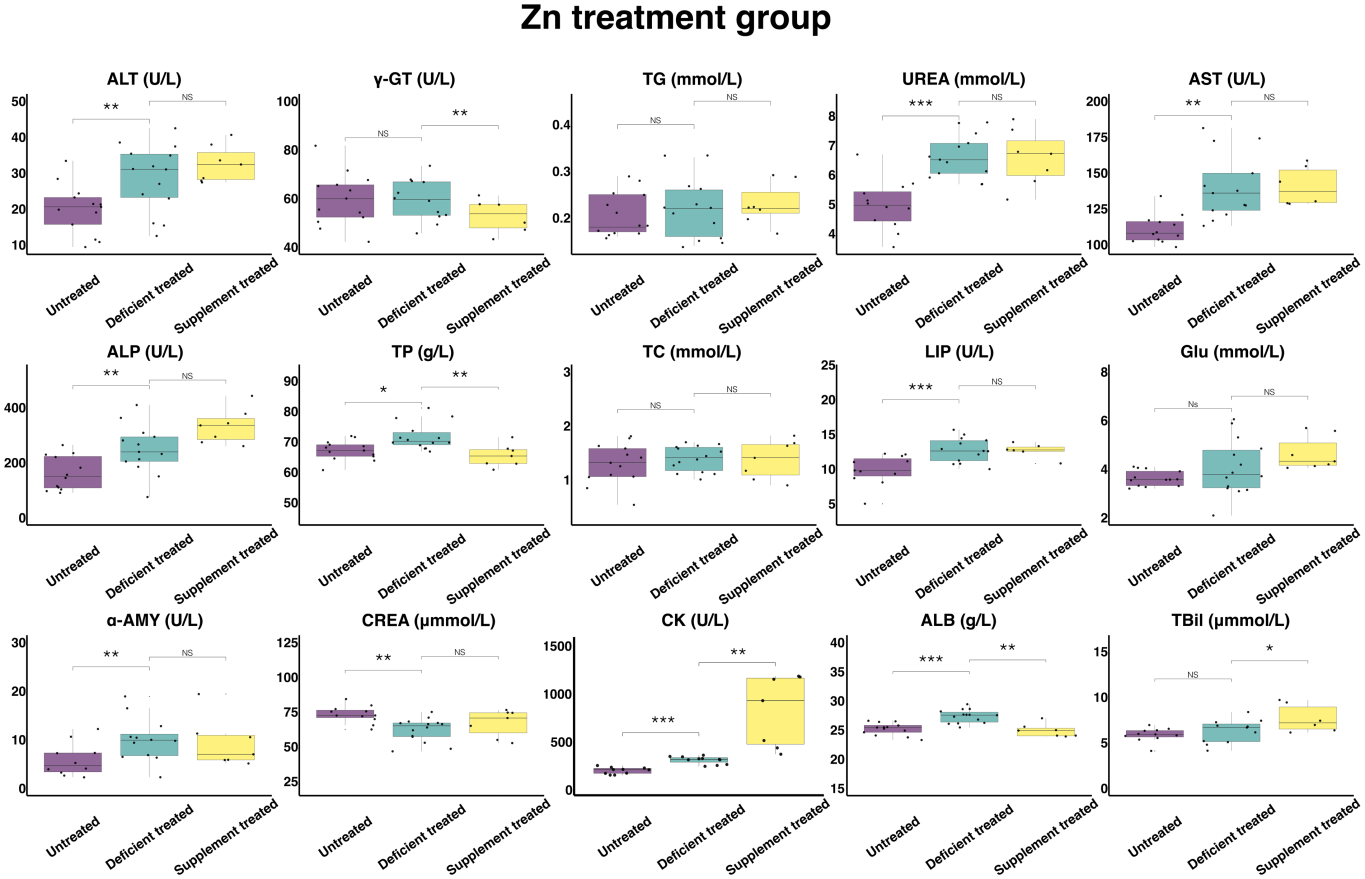
The results of the serum biochemical parameters concentration and the serum biochemical parameters activity in Zn treatment group. ^NS^*p* > 0.05, *p < 0.05, **p < 0.01, ***p < 0.001.

Figure 9 shows the results of serum biochemical parameters concentration and serum biochemical parameters activity in the Zn treatment group. The Zn deficiency treatment made no difference to the concentration of serum TG, TC, GLU and TBil and the activity of γ-GT. There was a significant increase in the concentration of ALB (27.36 ± 7.31 g/L vs. 25.19 ± 6.99 g/L, *p* < 0.05), UREA (6.61 ± 1.83 mmol/L vs. 4.97 ± 1.43 mmol/L, *p* < 0.05), TP (71.71 ± 19.17 g/L vs. 67.12 g/L ± 18.62 g/L, *p* < 0.05) and the activity of serum ALT (28.61 ± 7.65 U/L vs. 19.90 ± 5.52 U/L, *p* < 0.05), AST (140.1 ± 38.86 U/L vs. 110.79 ± 31.98 U/L, *p* < 0.05), ALP (247.66 ± 68.69 U/L vs. 162.06 ± 46.78 U/L, *p* < 0.05), LIP (12.75 ± 3.54 U/L vs. 9.9 ± 2.98 U/L, *p* < 0.05), α-AMY (10.03 ± 2.89 U/L vs. 5.86 ± 1.85 U/L, *p* < 0.05) and CK (298.29 ± 89.94 U/L vs. 189.84 ± 60.03 U/L, *p* < 0.05). In contrast, the concentration of the CREA (62.05 ± 16.58 umol/L vs. 73.28 ± 22.10 umol/L, *p* < 0.05) declined sharply compared with the untreated.

The Zn supplement treatment had no impact on the concentration of CREA, TG, UREA, TC, GLU or the activity of serum ALT, AST, ALP, LIP, α-AMY. The concentration of serum TBil (7.66 ± 3.13 umol/L vs. 6.55 ± 2.93 umol/L, *p* < 0.05) and the activity of serum CK (804.84 ± 304.20 U/L vs. 296.8 ± 121.17 U/L, *p* < 0.05) was significantly enhanced, while the concentration of serum TP (65.44 ± 24.74 g/L vs. 74.71 ± 28.24 g/L, *p* < 0.05) and ALB (24.91 ± 9.42 g/L vs. 27.87 ± 10.53 g/L, *p* < 0.05) and the activity of γ-GT (52.77 ± 21.54 U/L vs. 63.5 ± 25.92 U/L, *p* < 0.05) were suppressed significantly when compared with the Zn deficiency treatment.

#### 3.2.3. Concentration and activity of serum biochemical parameters in Cu treatment group

**Figure 10:**
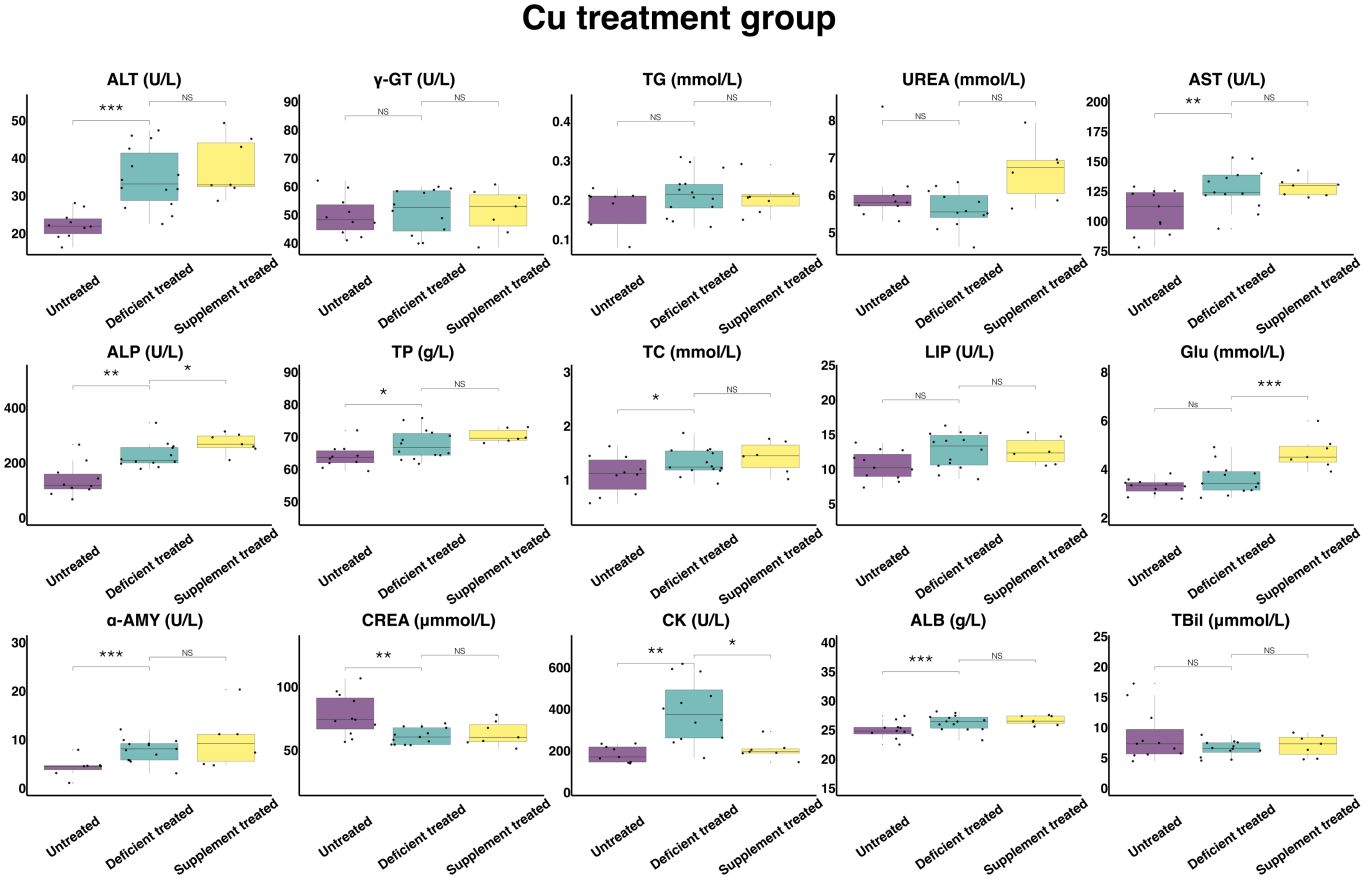
The results of the serum biochemical parameters concentration and the serum biochemical parameters activity in Cu treatment group. ^NS^*p* > 0.05, *p < 0.05, **p < 0.01, ***p < 0.001.

Figure 10 shows the results of serum biochemical parameters concentration and serum biochemical parameters activity in Cu treatment group. Following the Cu deficiency treatment, there was no difference shown by the concentration of serum TG, UREA, GLU, and TBil or the activity of γ-GT and LIP in comparison with the untreated period. There was a significant increase in the concentration of ALB (26.27 ± 7.02 g/L vs. 24.96 ± 7.53 g/L, *p* < 0.05), TP (67.74 ± 18.10 g/L vs. 63.99 ± 20.24 g/L, *p* < 0.05) and TC (1.33 ± 0.36 mmol/L vs. 1.10 ± 0.35 mmol/L, *p* < 0.05) and the activity of serum ALT (34.72 ± 9.28 U/L vs. 22.27 ± 7.04 U/L, *p* < 0.05), AST (127.48 ± 35.36 U/L vs. 107.89 ± 32.53 U/L, *p* < 0.05), ALP (228.13 ± 65.87 U/L vs. 138.56 ± 43.82 U/L, *p* < 0.05), α-AMY (7.83 ± 2.17 U/L vs. 4.34 ± 1.64 U/L, *p* < 0.05) and CK (391.07 ± 112.89 U/L vs. 183.72 ± 61.24 U/L, *p* < 0.05) when compared with the untreated period, and the concentration of only CREA (61.14 ± 16.96 umol/L vs.77.82 ± 23.46 umol/L, *p* < 0.05) was reduced.

Compared with the Cu deficiency treatment, the Cu supplement treatment made no difference to the serum concentration of TG, TBil, UREA, TP, TC, CREA, and ALB or the activity of ALT, γ-GT, AST, LIP, and α-AMY. The Cu supplement treatment significantly enhanced the concentration of GLU (4.70 ± 1.78 mmol/L vs. 3.40 ± 1.28 mmol/L, *p* < 0.05) and the activity of ALP (271.04 ± 102.44 U/L vs. 227.12 ± 92.72 U/L, *p* < 0.05) but suppressed the activity of CK (204.11 ± 77.15 U/L vs. 304.87 ± 124.46 U/L, *p* < 0.05) compared with the Cu deficiency treatment.

#### 3.2.4. Concentration and activity of serum biochemical parameters in Co treatment group

**Figure 11:**
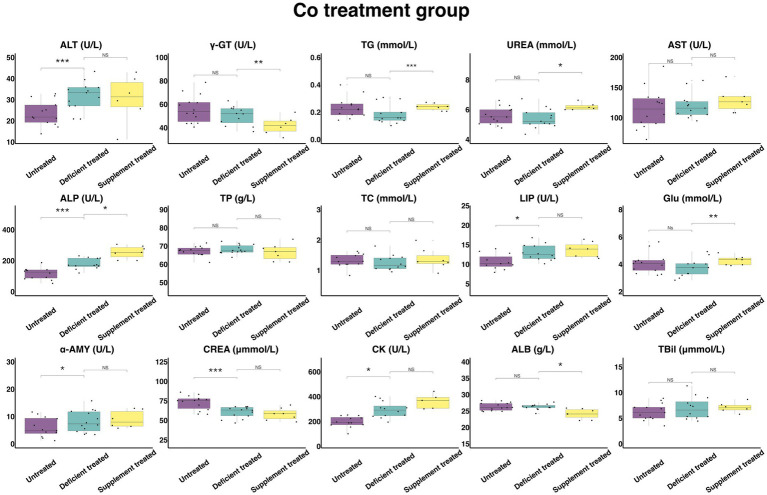
The results of the serum biochemical parameters concentration and the serum biochemical parameters activity in Co treatment group. ^NS^*p* > 0.05, *p < 0.05, **p < 0.01, ***p < 0.001.

Figure 11 shows the results of serum biochemical parameters concentration and serum biochemical parameters activity in Co treatment group. During the Co deficiency treatment, there were no significant differences observed in the concentration of TBil, TG, UREA, TP, TC, GLU, and ALB or the activity of γ-GT, AST when compared with the untreated. The concentration of CREA (60.51 ± 16.17 umol/L vs. 72.73 ± 19.44 umol/L, *p* < 0.05) was significantly reduced but the activity of ALT (31.79 ± 8.82 U/L vs. 23.58 ± 6.54 U/L, *p* < 0.05), ALP (179.24 ± 51.74 U/L vs. 115.95 ± 33.47 U/L, *p* < 0.05), LIP (12.97 ± 3.74 U/L vs. 10.65 ± 3.21 U/L, *p* < 0.05), α-AMY (8.2 ± 2.19 U/L vs. 6.05 ± 1.75 U/L, *p* < 0.05) and CK (293.44 ± 84.71 U/L vs. 197.97 ± 57.15 U/L, *p* < 0.05) was significantly enhanced in comparison with the untreated period.

During the Co supplement treatment, there was no significant difference shown by the concentration of TBil, TP, TC, and CREA, or the activity of the ALT, AST, LIP, α-AMY, and CK when compared with the Co deficiency treatment. Compared with the Co deficiency treatment, the concentration of TG (0.24 ± 0.090 mmol/L vs. 0.15 ± 0.061 mmol/L, *p* < 0.05), UREA (6.20 ± 2.53 mmol/L vs. 5.43 ± 2.22 mmol/L, *p* < 0.05) and GLU (4.28 ± 1.62 mmol/L vs. 3.31 ± 1.25 mmol/L, *p* < 0.05) and the activity of ALP (254.59 ± 96.22 U/L vs. 176.5 ± 72.06 U/L, *p* < 0.05) increased significantly but the concentration of ALB (24.16 ± 9.13 g/L vs. 25.96 ± 9.81 g/L, *p* < 0.05) and the activity of γ-GT (41.87 ± 17.09 U/L vs. 50.17 ± 20.48 U/L, *p* < 0.05) were reduced sharply after the Co supplement treatment.

#### 3.2.5. Concentration and activity of serum biochemical parameters in Mn treatment group

**Figure 12:**
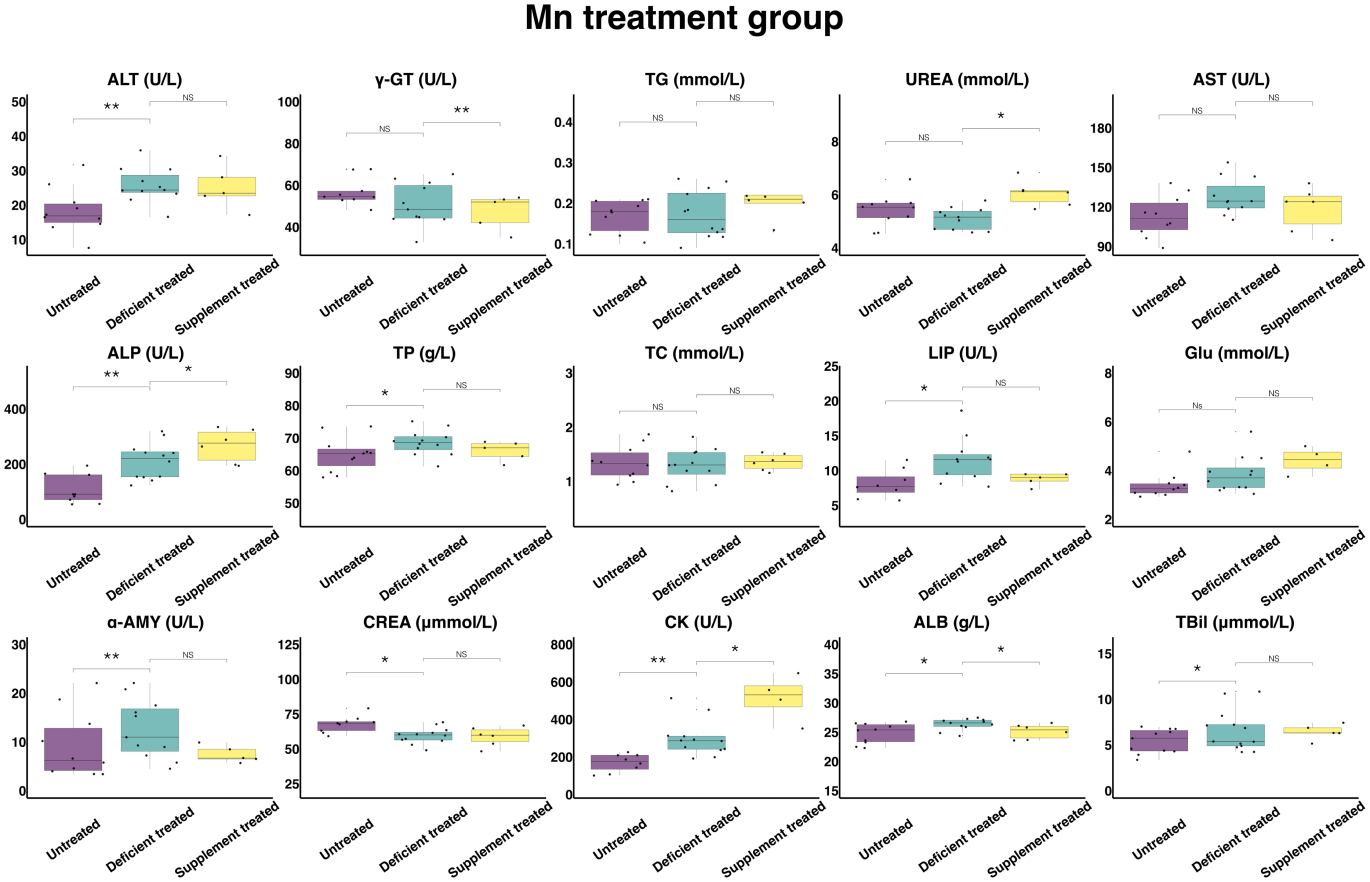
The results of the serum biochemical parameters concentration and the serum biochemical parameters activity in Mn treatment group. ^NS^*p* > 0.05, *p < 0.05, **p < 0.01, ***p < 0.001.

Figure 12 shows the results of serum biochemical parameters concentration and serum biochemical parameters activity in the Mn treatment group. There was no effect caused by Mn deficiency treatment on the concentration of TG, UREA, TC, and GLU and the activity of γ-GT and AST. There were not only a significant increase in the concentration of TBil (6.54 ± 1.81 umol/L vs. 5.42 ± 1.63 umol/L, *p* < 0.05), TP (68.58 ± 19.80 g/L vs. 64.92 ± 19.57 g/L, *p* < 0.05) and ALB (26.35 ± 7.95 g/L vs. 24.82 ± 7.85 g/L, *p* < 0.05) and the activity of ALT (25.76 ± 7.77 U/L vs. 18.34 ± 5.80 U/L, *p* < 0.05), ALP (210.73 ± 60.83 U/L vs. 107.63 ± 35.88 U/L, *p* < 0.05), LIP (11.58 ± 3.49 U/L vs. 8.1 ± 2.86 U/L, *p* < 0.05), α-AMY (12.6 ± 3.80 U/L vs. 9.25 ± 2.93 U/L) and CK (298.43 ± 86.15 U/L vs. 168.65 ± 59.63 U/L, *p* < 0.05), but also a sharp decline in the concentration of CREA (59.57 ± 17.20 umol/L vs. 67.78 ± 22.59 umol/L, *p* < 0.05) when compared with the untreated period.

Mn supplement treatment had no effect on the concentration of TBil, TP, TC, TG, GLU, and CREA or the activity of ALT, AST, LIP, and α-AMY when compared with the Mn deficiency treatment. This treatment caused not only a significant increase in the concentration of UREA (6.055 ± 2.47 mmol/L vs. 4.85 ± 2.17 mmol/L, *p* < 0.05) and the activity of CK (515.48 ± 257.74 U/L vs. 236.23 ± 96.44 U/L, *p* < 0.05) and ALP (267.42 ± 109.17 U/L vs. 192.88 ± 78.74 U/L, *p* < 0.05), but also a decrease in the concentration of ALB (25.13 ± 10.26 g/L vs. 26.8 ± 11.99 g/L, *p* < 0.05) and the activity of the γ-GT (47.3 ± 21.15 U/L vs. 55.04 ± 24.61 U/L, *p* < 0.05) in comparison with the Mn deficiency treatment.

#### 3.2.6. Concentration and activity of serum biochemical parameters in Se treatment group

**Figure 13:**
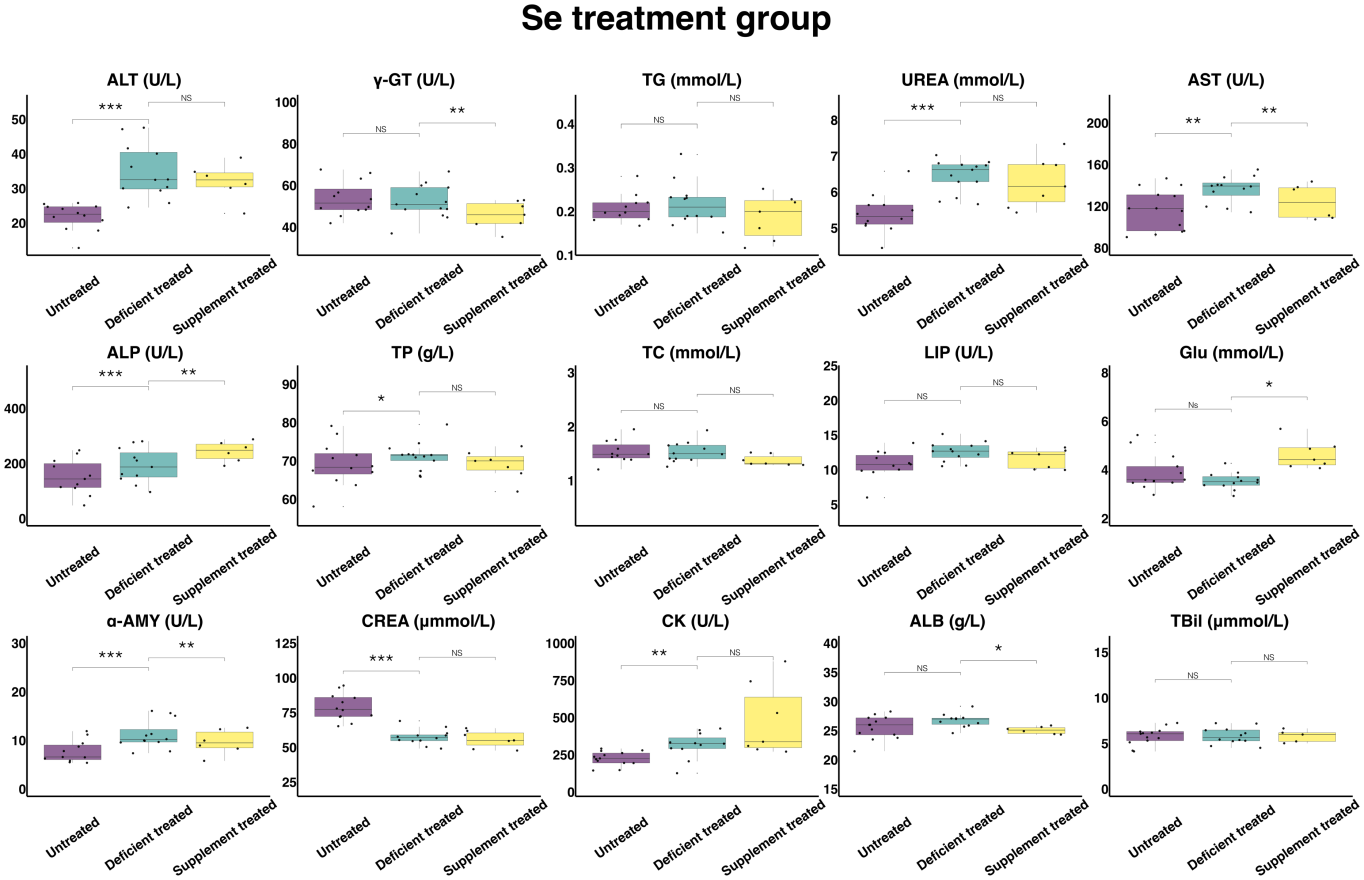
The results of the serum biochemical parameters concentration and the serum biochemical parameters activity in Se treatment group. ^NS^*p* > 0.05, *p < 0.05, **p < 0.01, ***p < 0.001.

Figure 13 shows the results of serum biochemical parameters concentration and serum biochemical parameters activity in Se treatment group. Se deficiency treatment had no effect on the concentration of TBil, TG, TC, GLU, and ALB or the activity of γ-GT, LIP. After Se deficiency treatment, the concentration of UREA (6.44 ± 1.79 mmol/L vs. 5.40 ± 1.56 mmol/L, *p* < 0.05), TP (71.19 ± 19.75 g/L vs. 69.14 ± 19.96 g/L, *p* < 0.05) and the activity of ALT (34.83 ± 10.06 U/L vs. 21.79 ± 6.29 U/L, *p* < 0.05), AST (136.23 ± 39.32 U/L vs. 116.72 ± 32.37 U/L, *p* < 0.05), ALP (192.01 ± 57.89 U/L vs. 150.99 ± 45.53 U/L, *p* < 0.05), α-AMY (11.15 ± 3.22 U/L vs. 7.74 ± 2.33 U/L, *p* < 0.05) and CK (314.30 ± 94.77 U/L vs. 225.56 ± 62.56 U/L, *p* < 0.05) were significantly enhanced but the concentration of CREA (57.49 ± 16.60 umol/L vs. 78.90 ± 22.78 umol/L, *p* < 0.05) was reduced compared with the untreated period.

Se supplement treatment made no difference to the concentration of TBil, TG, UREA, TP, TC and CREA or the activity of the ALT, LIP and CK. After Se supplement treatment, the concentration of GLU (4.63 ± 1.75 mmol/L vs. 3.56 ± 1.35 mmol/L, *p* < 0.05) and the activity of ALP (243.42 ± 99.37 U/L vs. 172.27 ± 70.33 U/L, *p* < 0.05) increased significantly but the concentration of ALB (25.07 ± 10.23 g/L vs. 26.37 ± 10.76 g/L, *p* < 0.05) and the activity of γ-GT (45.73 ± 17.28 U/L vs. 54.87 ± 20.74 U/L, *p* < 0.05), AST (124.28 ± 50.74 U/L vs. 136.96 ± 51.76 U/L, *p* < 0.05) and α-AMY (9.67 ± 3.95 U/L vs. 13.12 ± 5.35 U/L, *p* < 0.05) decreased significantly compared with the Se deficiency treatment.

## 4. Discussion

Mineral elements are the essential nutrients needed by ruminants. The appropriate concentration of essential mineral elements enables the normal development of their physiological, catalytic, and regulatory functions (39). Although many researches indicated plasma samples were more suitable for measuring concentrations of mineral Ca, P, S, and Cu, there were many studies using serum samples to detected these mineral elements and established ranges of the standard concentration for sheep (15, 40–43). To uniform this study, the serum samples were used to measure both the concentration of the mineral elements and the biochemical parameters. It was found out that the normal serum Ca concentration in junior sheep ranged from 70 mg/L to 80 mg/L (3). In this research, the mean value of the initial concentration of Ca reached 51.61 mg/L, indicating that the Ca deficiency found in Mongolian Wu Ranke sheep resulted from their long-term pattern of grazing in the grassland. Serum Ca usually does not fluctuate with the concentration of Ca in the diet (42). After 60 days of Ca deficiency treatment, there was only an insignificant decrease observed in the concentration of serum *Ca.* Similarly, there was no significant increase found in the other treatment group’s serum Ca concentration after Ca supplement was treated based on the addition of NRC-recommended. Bone tissues, which are intended to maintain Ca homeostasis in the blood and extracellular fluid, play a major role in Ca storage for animals (42). When animals are severely deficient in Ca, the body shows an increase in bone resorption to maintain serum Ca concentration as normal. Eventually, the mineral concentration in bone tissues is reduced to the minimum, which impairs bone mineral deposition (44). Therefore, bone health is worthy of attention for the Mongolian Wu Ranke sheep with low serum Ca concentrations. Although serum Ca concentration does not change immediately with the level of dietary Ca concentration, the concentration of serum Ca in the Ca supplement group still increased significantly from 47.69 mg/L to 61.98 mg/L after 41 days of dietary Ca supplementation. Interestingly, the level of serum Ca concentration was not significantly improved in the other treatment groups during the 101-day rearing period. This may be due to the sheep with Ca deficiency requiring s large-scale Ca supplementation for the change in serum concentrations. The serum P and Ca have a combined effect on bone health for animals (42). There was a metabolic correlation discovered between mineral elements, and the concentration of serum Ca has affected the utilization of serum P. As revealed by the previous research, a diet with high P concentrations increased serum P concentration but decreased serum Ca concentration, suggesting a negative correlation (45). Our research also indicated that low Ca diets reduced serum Ca concentration but increased serum P concentration to a significant extent. Notably, there was a significant increase in serum P concentration but a sharp decline in serum Ca concentration after Mn supplement treatment, which is suspected to result from the dietary Mn promoting the absorption of serum P and thus decreasing the level of serum Ca concentration. Therefore, the ratio of dietary P to Mn must be considered to avoid reduced Ca absorption. The concentration of serum P in healthy sheep ranged from 46.5 mg/L to 58.9 mg/L (36). In comparison, the initial serum P concentration of the 84 Mongolian Wu Ranke sheep was 50.77 mg/L, which indicates that the concentration of P derived from the pasture could meet their nutritional needs. Therefore, the supplementation of dietary P is not required for those Mongolian Wu Ranke sheep.

As one of the essential mineral elements, Zn plays an important role in various life activities (46). The concentration of serum Zn in healthy sheep ranged from 0.6 mg/L to 1.2 mg/L (36). Comparatively, the mean value of initial serum Zn concentration was 0.65 mg/L in these 84 Mongolian Wu Ranke sheep studied. It is indicated that these Mongolian Wu Ranke sheep were not deficient in Zn. There was no decrease observed in serum Zn concentration after Zn deficiency treatment, which is consistent with the result of some other research (47). This may be attributable to the concentration of Zn in oats and natural pastures meeting the need of sheep for Zn. Similarly, after 41 days of Zn supplementation, there was no significant increase found in serum Zn. Usually, Zn is involved in the generation of metallothionein and gene regulatory proteins (48). The diets with high Zn concentrations can enhance metallothionein production in mucosal cells. When mucosal cells are shed, Zn returns to the gastrointestinal tract, thus inhibiting Zn absorption (49). Moreover, due to the inhibitory mechanism of high Zn, the supplementary dietary Zn was not fully absorbed by the Mongolian Wu Ranke sheep in this research (49). Interestingly, there was a sharp decrease in serum Cu concentrations after 41 days of Zn supplement, which is coherent with the result of many other studies (47, 50). This may also result from the high affinity of copper for metallothionein. In the high Zn state, mucosal cells return Cu to the gastrointestinal tract when shed (49, 51). Competing for the binding sites on enzymes, metalloproteins and metal transporter proteins, Cu and Zn produce antagonistic effects to some extent (52).

Although Cu deficiency can occur in all mammals, it is more common in ruminants (53). This is because Fe reacts with sulfide in the rumen to form the Fe-S complexes that can get dissociated from the abomasum to form insoluble CuS, thus reducing the uptake of Cu (54). The initial concentration of serum Cu was 0.57 mg/L in the 84 Mongolian Wu Ranke sheep. In the previous research, the serum Cu concentration was found to range from 0.70 mg/L to 1.36 mg/L in healthy sheep, indicating that the Mongolian Wu Ranke sheep under study developed Cu deficiency (36). The feeding on Cu deficient diet for 60 days significantly reduced serum Cu concentration to 0.46 mg/L, which suggests that dietary Cu content can reflect serum Cu concentration. The serum Cu concentration rose sharply to 0.61 mg/L after 41 days of Cu supplementation. Thus, long-term dietary Cu supplementation is recommended for the Mongolian Wu Ranke sheep as the accumulation of Cu in sheep may be instantaneous. In this research, the concentration of serum Cu in the Ca, Zn, Co, Mn, and Se treatment groups showed a decreasing trend after 101 days of feeding on the diets containing multi-nutrient salts, which may result from serum Fe and serum Cu competing for the binding sites on intestinal proteins, with excess Fe inhibiting the absorption of Cu (55). The mean value of initial serum Fe concentration in these 84 Mongolian Wu Ranke sheep studied was 2.03 mg/L. By comparison, the serum Fe concentration in healthy sheep was in the range of 1.00 mg/L to 2.00 mg/L (36). On day 101 of feeding with multi-nutrient mineral salts, the serum Fe concentration in these 84 Mongolian Wu Ranke sheep increased to 4.80 mg/L, which is beyond the normal range. For the sheep, serum Cu and Fe showed a significant negative correlation, with excess Fe decreasing the Cu concentration in the sheep serum (56). At the same time, the multi-nutrient mineral salts in the diet contained S, and serum S concentration gradually increased in the blood collected three times. This also provided elements Fe and S required for the formation of Fe-S complexes in the rumen, which reduces Cu absorption. It is demonstrated that Mongolian Wu Ranke sheep have sufficient Fe, which means no need for dietary Fe supplementation.

Mn is involved in energy metabolism and is effective in promoting the utilization of fat (32). The mean value of the initial concentration of serum Mn was 0.033 mg/L. According to some research, the serum Mn concentration in healthy sheep was 0.04 mg/L to 0.05 mg/L, which indicates a slight deficiency of Mn in the sheep studied (36). The deficiency of Ca and Mn could impede bone formation and thus reduce the growth rate for animals (42, 57). Therefore, it is worth paying attention to the skeletal health of the Mongolian Wu Ranke sheep regularly. The serum Mn concentrations in the Mn treatment group decreased after 60 days of feeding on the Mn deficiency diet and then increased after 41 days of feeding on the Mn supplement diet. The mean value of serum Mn concentration was 0.094 mg/L, 0.10 mg/L, 0.056 mg/L, 0.045 mg/L and 0.11 mg/L in the Ca, Zn, Cu, Co, and Se treatment groups after 60 days of feeding with multi-nutrient mineral salts, which shows that Mn can be replenished in the serum of Mongolian Wu Ranke sheep through the prompt supplementation of Mn in the diet.

Among all of the measured essential mineral elements, serum Se deficiency was found most severe in this study. The serum Se concentration in healthy sheep was determined to be in the range of 0.09 mg/L to 0.50 mg/L (36). However, the initial concentration of serum Se was merely 0.015 mg/L in the sheep studied, which indicates Se deficiency. Although the ruminants with Se deficiency may suffer from leukodystrophy, the Mongolian Wu Ranke sheep in the study showed no clinical signs, such as hair loss or muscular dystrophy (5, 58). Due to the strong reducing environment in the rumen of ruminants, selenium was partially converted into insoluble compounds, which is adverse to the Se storage (59). Therefore, timely Se supplementation is required for the sheep. After 60 days of feeding with multi-nutrient salts, the serum Se concentration in Se deficiency treatment exceeded the lower limit of detection and could not be measured. The mean value of serum Se concentration in Ca, Zn, Cu, Co, and Mn deficiency groups increased significantly to 0.087 mg/L, 0.083 mg/L, 0.091 mg/L, 0.090 mg/L, and 0.082 mg/L, which falls within the normal range as recommended. This result is similar to that of some other research (60), indicating that serum Se can be used to indicate the short-term Se status of the sheep. In spite of this, long-term dietary supplementation of Se is still recommended. However, dietary mineral element status may not be the only factor affecting the concentration of serum mineral elements. They are also influenced by the animal species, individual animal variability, metabolic processes and interactions between mineral elements (55).

Serum biochemical parameters are closely related to a range of physiological processes such as animal performance and lipid metabolism (61, 62). These parameters can be used to effectively assess animals for their nutritional status, tissue damage and organ dysfunction. ALT (63), AST (63), ALP (64), γ-GT (65), T-Bil (66), ALB (67), and TP (68) are applicable to evaluate the animal for liver function or impairment. In addition, ALP is a reliable indicator of bone mineralization identification. Abnormal ALP activity can disrupt the dynamic balance inside animals through certain factors. When exceeding the normal levels, ALP activity may also indicate the development of many diseases, such as excessive bone mineralization, tumors, and potentially Alzheimer’s disease (69). In the research, it is indicated that the normal activity of sheep serum ALP varied from 70 U/L to 390 U/L (70). The activity of ALP of all the Mongolian Wu Ranke sheep studied fell within the normal range for the three serum collections. The broiler fed with Ca-deficient diet showed increased ALP activity, which impairs bone development (71). The level of serum Ca concentration in this study was usually low Due to the enhanced serum ALP activity and insufficient Ca concentration, the skeletal development of the Mongolian Wu Ranke sheep may be hindered.

ALT accumulates mainly in the cytoplasm of hepatocytes, in which the ALT activity is approximately 3,000 times stronger than in serum. Therefore, when the liver suffers damage, the damaged hepatocytes release ALT, which significantly enhances the serum ALT activity (72). As a cytoplasmic and mitochondrial enzyme used to catalyze the reversible reaction of aspartate deamination to oxaloacetate, AST can participate in the Krebs cycle. AST elevation may occur in hepatocyte or muscle damage diseases (73). In the context of liver injury, fatty liver and hepatitis, serum AST and ALT activity are significantly increased. It has been shown that the ALT and AST activities increased significantly in Ca-deficient rats (74). In the Ca deficiency treatment, the activities of serum AST and ALT increased significantly, as did the concentrations of ALB and T-Bil. ALB is produced mainly by the liver and the rise of ALB concentration may be associated with liver dysfunction (67). As a breakdown product of hemoglobin, TBil can be used as a diagnostic indicator of liver and blood disorders. TBil can be divided into non-conjugated bilirubin and conjugated bilirubin. Elevated non-conjugated bilirubin can lead to liver damage and dysfunction, thus causing hyperbilirubinemia (66). The elevation of these indicators indicates a possibility that feeding on Ca-deficient diet causes liver damage in the sheep studied. In Ca supplement treatment, there were no significant differences observed in serum AST, ALT, ALB, and T-Bil when compared with Ca deficiency treatment, which may suggest that the dietary Ca supplementation for 41 days failed to alleviate liver damage or dysfunction. In the research, it was indicated that the Cu deficiency in Kazakh sheep caused a significant increase in ALT and AST activities, which resulted in hepatocyte damage (13). The same results were also found in the sheep serum in the Cu deficiency group. TP is an important indicator of liver damage (68). A significant increase in the activity of AST, ALT and the concentration of ALB, TP may also indicate that Cu deficiency contributed to hepatocyte damage for the Mogolian Wu Ranked sheep. However, there was no significant reduction observed in these four indicators among the Cu supplement group, which may be due to the ineffectiveness of short-term dietary Cu supplementation in reducing the liver damage caused by Cu deficiency. Serum ALT activity was significantly elevated in Co deficiency treatment indicating that Co deficiency may have a negative effect on the liver. However, there was a significant decrease in serum ALB concentration and γ-GT activity after feeding on Co supplementation diet. The enhancement of serum γ-GT activity was associated with liver damage. Due to γ-GT elevation, glutathione was degraded to cysteine-glycine, which led to the generation of ROS. By inhibiting the elevation of γ-GT activity, cellular oxidative stress and membrane damage can be prevented (75). Although Co was not detected in serum in the study, the results of serum biochemical parameters still showed that Co supplementation could contribute to alleviating the liver damage caused by Co deficiency. In general, the increase in serum ALT and AST activity can be referenced to diagnose the Se deficiency in sheep (5). Se deficiency caused a significant increase in the activity of serum AST, ALT, and concentration of TP for the Mongolian Wu Ranke sheep, thus resulting in the impairment of liver function. Following the dietary supplementation with Se, Se supplement treatment significantly suppressed the activity of AST, γ-GT and the concentration of ALB, which may demonstrate that timely Se supplement can reduce the value of liver-related parameters and promote liver function. After 60 days of feeding on Mn deficiency diet, there was a significant increase found in the concentration of TBil and ALB and the activity of ALT, which adversely affects liver function. For the Mn supplement treatment, the serum γ-GT activity and ALB concentration decreased significantly after the Mn supplement. Previous research showed that Mn supplement significantly inhibited AST and ALT activity in rats (74). Despite no significant differences in Mn supplement treatment. ALT and AST, γ-GT, and ALB can also be taken as the indicators of liver function. It is suggested that Mn supplement may have a beneficial effect on the liver of the Mongolian Wu Ranke sheep. A significant increase in the concentration of ALB, TP and the activity of AST, ALT may also indicate that Zn deficiency contributed to hepatocyte damage for the Mongolian Wu Ranked sheep. Following the dietary supplementation with Zn, Zn supplement treatment significantly suppressed the concentration of ALB and TP and the activity of γ-GT, which may demonstrate that timely Zn supplement could contribute to alleviating the liver damage caused by Zn deficiency.

In addition to impairing liver function, the deficiency in dietary essential mineral elements can also cause damage to the skeletal muscle or myocardium of the sheep. CK is known as one of the most abundant intracellular enzymes in skeletal muscle and myocardium, and serum CK serves as a good indicator of skeletal muscle and myocardium injury. Besides, the elevation of serum CK activity has been accepted as a means to detect myocardial infarction (76). In the research, it is shown that serum CK elevation was exhibited by the sheep clinically or subclinical infected with leukodystrophy (59). However, the Mongolian Wu Ranke sheep with Se deficiency diet exhibited neither clinical nor subclinical leukodystrophy. The concentration of serum CK in Cu supplement treatment decreased significantly after Cu supplementation, which may imply the effectiveness of Cu supplement in alleviating the damage caused to skeletal muscle or myocardium.

TC and TG are used as the parameters of lipid metabolism in serum biochemical analysis (61). TC concentrations were found significantly higher in Cu deficiency treatment. The decrease in serum Cu concentration may be partly responsible for the increase in TC concentration. The intracellular concentrations of reduced glutathione elevation can enhance the activity of cholesterol synthesis rate-limiting enzymes. If the cellular concentration of reduced glutathione increases due to Cu deficiency, then the activity of rate-limiting enzymes increases as well, thus enhancing cholesterol synthesis (55). The two important components of TC are high-density lipoprotein and low-density lipoprotein. By affecting lipid metabolism, excess TC leads to metabolic disorders. TG refers to a fat molecule formed by the condensation of long-chain fatty acids and glycerol. Produced by the oxidative breakdown of fats, ATP can provide energy to most tissues (61). It has been demonstrated that the concentration of TC is correlated with that of TG. TG concentration was found higher in junior sheep and TC concentration was found irrelevant to the age of sheep (77). In the research, a positive correlation was discovered between serum Ca and TG concentrations (78). The TG concentration of the sheep was significantly lower in Ca deficiency treatment but significantly higher in Ca supplement treatment, which is consistent with the result of some other research. Also, dietary Co supplements increased the level of serum TG concentrations, which shows the possibility of dietary Ca and Co supplements increasing the body fat content in the Mongolian Wu Ranke sheep. Interestingly, serum LIP activity was enhanced significantly in Ca deficiency treatment. LIP can help digest fat and the increase in LIP activity can decrease TG concentration (79). This translates into a decrease in serum TG concentration for Ca deficiency. LIP and α-AMY are the biomarkers of pancreas injury, the elevation of which provides a diagnostic basis for acute pancreatitis (80). In the research, it is shown that Mn deficiency caused a significant increase in α-AMY activity, which is consistent with the results of this study (81). According to the results of serum LIP and α-AMY, such mineral elements as Ca, Zn, Cu, Co, Mn, and Se deficiency may impair pancreas function for the Mongolian Wu Ranke sheep. After 41 days of Se supplementation, the α-AMY activity decreased significantly in Se supplement treatment. It may be attributed to the effectiveness of dietary Se supplementation in restoring pancreas function.

In normal circumstances, serum GLU concentration is 2.8 mmol/L to 5.6 mmol/L for sheep (60). The serum GLU concentration of all the Mongolian Wu Ranke sheep studied fell within the normal limits, with no hypoglycemia or hyperglycemia detected. However, there was a significant increase observed in GLU concentration for the Wu Ranke sheep receiving Ca, Cu, Co and Se supplement treatments. It has been shown that the dietary Co and Se supplementation for sheep can increase glucose concentrations (59, 60, 82). It may be because propionic acid is the primary precursor of glucose in ruminants. For sheep, Co supplementation led to a significant increase in propionic acid concentrations, while the higher propionic acid concentrations resulted in higher serum glucose concentrations (17). This is coherent with the finding of the previous research, which may indicate that the dietary supplementation of Ca, Cu, Co, and Se can improve the generation of energy for the Mongolian Wu Ranke sheep.

CREA and UREA are commonly used as biomarkers of renal function. The kidneys are responsible for various important physiological functions, such as filtering blood and removing toxins (83). As a kind of metabolic waste, CREA is mainly excreted by the kidneys. When the kidney is dysfunctional, the level of serum CREA concentration rises sharply (84). As the main nitrogen metabolite in the blood, UREA is derived mainly from the degradation of proteins. Its content is higher in adult sheep than in junior sheep (77). However, the elevation of UREA and CREA does not necessarily indicate the development of kidney disease. This is considered to be normal within the standard range (85). The normal range of CREA and UREA in sheep is 44.2 umol/L–132.6 umol/L and 2.8288 mmol/L–9.984 mmol/L, respectively (86). The concentration of serum CREA and UREA was within the normal range for all the Mongolian Wu Ranke sheep studied, indicating that the kidney function of the Mongolian Wu Ranke sheep was unaffected by mineral element deficiency and supplementation. In addition, there are still many limitations in this study. Firstly, the feeding experiments might be better to design 30, 60, and 90 days to obtain more reasonable days of supplementation for grazing sheep. In this research, the effects of essential mineral element deficiency and supplementation on the liver, myocardium and pancreas were determined only by serum levels of mineral elements and biochemical parameters. In the future, morphological, pathological and biochemical parameters might be explored directly on the liver, myocardium and pancreas to further validate the results from this research.

In conclusion, according to the results of the serum essential mineral element measurement, the grazing Mongolian Wu Ranke sheep in this study developed Ca, Cu, Co, Mn, and Se deficiencies. During the 60 days of experiment on dietary Ca, Zn, Cu, Co, Mn, and Se deficiency, the serum biochemical parameters showed that the deficiency of the essential mineral elements was damaging to the liver of the Mongolian Wu Ranke sheep, and their myocardium and pancreas functions. In the 41 days of dietary Ca, Zn, Cu, Co, Mn, and Se supplements after the deficiency experiment, the supplementation with Ca, Cu, and Se significantly increased the level of serum Ca, Cu, and Se concentrations. The dietary supplementation of Zn, Co, Mn, and Se reduced liver-related indicators and alleviated liver damage. The supplementation of Cu possibly restored myocardium function. Se supplementation reduced pancreas parameters. The supplementation of Ca and Co possibly increased the fat content. Ca, Cu, Co, and Se supplementation increased the serum glucose concentration and possibly improved the generation of energy. To sum up, serum elements and biochemical parameters measurement indicated that the prompt supplementation of essential mineral elements like Ca, Zn, Cu, Co, Mn, and Se is required for grazing Mongolian Wu Ranke sheep to avoid the damage caused to their liver, myocardium and pancreas. This study provided data guidance for rational mineral supplementation in grazing sheep, which can help improve the overall health and productivity of grazing sheep and further promote increased production and income of herders.

## Data availability statement

The original contributions presented in the study are included in the article/Supplementary material, further inquiries can be directed to the corresponding author.

## Ethics statement

The animal study was reviewed and approved by Inner Mongolia University Animal Care and Use Committee.

## Author contributions

XJ: writing—original draft. LMe: data curation. RZ: validation. MT: visualization. ZQ: conceptualization. LMi: writing—review and editing. All authors contributed to the article and approved the submitted version.

## Funding

This research was funded by the Research Foundation for Advanced Talents of Inner Mongolia University.

## Conflict of interest

The authors declare that the research was conducted in the absence of any commercial or financial relationships that could be construed as a potential conflict of interest.

## Publisher’s note

All claims expressed in this article are solely those of the authors and do not necessarily represent those of their affiliated organizations, or those of the publisher, the editors and the reviewers. Any product that may be evaluated in this article, or claim that may be made by its manufacturer, is not guaranteed or endorsed by the publisher.
