# Supplementary material for: Effects of essential mineral elements deficiency and supplementation on serum mineral elements concentration and biochemical parameters in grazing Mongolian sheep
Source: Front Vet Sci. 2023 Jul 25;10:1214346. doi: 10.3389/fvets.2023.1214346 (PMC10407109; doi:10.3389/fvets.2023.1214346)
Supplement: Supplementary file 1 [file Table_1.docx]

Supplementary Material

Effects of Essential Mineral Elements Deficiency and Supplementation on Serum Mineral Elements Concentration and Biochemical Parameters in Grazing Mongolian Sheep

# Supplementary Table

Table S1. The digestion procedure of the microwave digester.

| Steps | Control temperature  (℃) | Heating time  (min) | Constant temperature time  (min) |
| --- | --- | --- | --- |
| 1 | 120 | 5 | 5 |
| 2 | 150 | 5 | 10 |
| 3 | 190 | 5 | 20 |
